# Inverse Estimation of Moisture Diffusion Model for Concrete Using Artificial Neural Network

**DOI:** 10.3390/ma15175945

**Published:** 2022-08-28

**Authors:** Jae Min Lee, Chang Joon Lee

**Affiliations:** Department of Architectural Engineering, Chungbuk National University, Cheongju 286442, Korea

**Keywords:** inverse estimation, concrete moisture diffusion model, artificial neural network (ANN)

## Abstract

In this research, the moisture diffusion model for concrete was inversely estimated using artificial neural network (ANN) and the data collected from virtual experiments. In addition, the moisture distribution was predicted using the ANN model in numerical analysis. For inverse estimation, virtual experimental data were used. The virtual experimental data were generated by adding noise to the moisture distribution obtained by a numerical simulation using a known moisture diffusion model. ANNs of two architectures were used in the inverse estimation. For performance test, the inversely estimated ANN model and the known moisture diffusion model were compared. The predicted humidity distribution using the ANN and virtual experiment data were also compared. The inversely estimated ANN model was in a good agreement with the known moisture diffusion model used for the virtual experiment.

## 1. Introduction

When concrete is exposed to ambient after casting, moisture gradient inside concrete occurs due to the moisture evaporating from the concrete surface, and it leads to diffuse moisture in concrete. Moisture diffusion of concrete causes differential moisture distribution in concrete. Moisture distribution in concrete affects mechanical properties, long-term behavior, durability, etc. For example, dry concrete has a lower elastic modulus than saturated concrete, because water in the pores is incompressible and cannot escape quickly when the concrete is subjected to rapid loading [1]. As the moisture content in concrete is consumed in hydration process, the degree of hydration increases and porosity decreases. The high degree of hydration and the low porosity promote early strength development [2]. Drying shrinkage caused by differential moisture distribution with position creates cracks, and as cracks are evolved by external loads, performance of concrete structure can be degraded [3]. When relative humidity in pores increases, long term creep rate and deflection of concrete member also increase. The deflection of concrete member cannot ensure serviceability of concrete structures [4,5,6]. The relative humidity around 75% increases the rate of carbonation. Carbonation degrades the durability of reinforced concrete as it leads to corrosion of reinforcing steel [7,8]. Through these examples, one may say that the humidity distribution affects properties and behaviors of the concrete, hence the prediction of the humidity distribution is an important issue to understand the mechanical and chemical behavior of concrete materials.

To predict the moisture distribution in concrete through numerical analysis, a moisture diffusion model is needed. Bazant and Najjar [9] suggest a moisture diffusion model for concrete considering pore relative humidity and temperature based on moisture diffusion theory. Mihashi and Nuamo [10] and Kang [11] proposed a moisture diffusion model in which the term for temperature was modified, and porosity with age was considered and modified, respectively. In addition, Sakata [12] suggests a moisture diffusion model represented by water content. These models have been used widely for moisture diffusion analysis of concrete.

The moisture diffusion models have a specific mathematical form with some model parameters. The model parameters hold some physical meanings, and are affected by water/cement ratio, aggregate type, curing methods, etc. The model parameters are commonly estimated using the moisture distribution data obtained through controlled laboratory tests, hence the information defining the test material is well known. The information helps to estimate the moisture diffusion model of the material. However, in the case of concrete materials without such information, there is a problem in estimating the moisture diffusion model. Incomplete information makes the inverse estimation of the material parameters unstable. In this case, the inverse estimation based on artificial neural network (ANN) can be considered as a good alternative.

ANN is one of the machine learning techniques for data analysis. ANN has functional non-uniqueness and imprecision tolerance. The functional non-uniqueness is a property which means that the internal structure of ANN can vary depending on training data. The imprecision tolerance is the property that ANN is robust to error such as noise [13]. Recently, various machine learning techniques including ANN have been used in characterization and optimization of cement-based materials [14,15]. The ANN has also been utilized in inverse modeling of material behavior for non-linear elastic materials [16], rate-dependent material [17], time-dependent structures [18], non-linear thermal material [19], etc. The benefit of using an ANN for these applications is that the ANN has functional flexibility, i.e., ANN can map arbitrary nonlinear relationships between input and output data.

In inverse estimation of material behavior, one may use a specific form of mathematical function that can capture the actual material behavior only if it is known that the material behavior obeys the specific form of mathematical function. However, in general, it is not reasonable to limit the material behavior to a specific form of mathematical function simply because the material behavior is unknown. If one uses a specific form of mathematical function as a material model, the inversely estimated material behavior is restricted by the capability of the mathematical function. For example, if a linear function is used as a material model, the inversely estimated material behavior always shows a linear behavior even when the system has highly non-linear material, so the material model estimation and prediction may be irrational. Using ANN as a material model in inverse estimation could be one of the solutions in that ANN material model does not restrict to a specific form of relationship but can map arbitrary nonlinear relationships between input and output data.

The purpose of this study is to inversely estimate the moisture diffusion model using ANN and moisture distribution data from a virtual experiment. The moisture diffusion theory is presented with moisture diffusion equation and moisture diffusion model. A finite element formulation for the moisture diffusion equation is presented. A procedure for the inverse estimation of moisture diffusion model using ANN is also presented. For performance tests, inversely estimated ANN model and a known moisture diffusion model were compared. The humidity distribution in concrete predicted by finite element simulation using the ANN model was also compared with that provided by the virtual experiment.

## 2. Moisture Diffusion Analysis of Concrete

### 2.1. Moisture Diffusion Equation

Diffusion is the process by which matter is transported from one part of a system to another as a result of random molecular or ion motions and is described by Fick’s first and second law [20]. According to Fick’s first law, the rate of diffusing substances in a steady state is proportional to the concentration of matter on each location. A diffusion flux due to moisture diffusion in concrete is represented using the pore relative humidity and water content and expressed using the pore relative humidity as Equation (1).

(1) $$J=-C_{k}grad\;H\;\left(1\right)$$

in which $C_{k}$ is permeability and H is the pore relative humidity.

Through Fick’s second law, mass balance equation in a transient state can be expressed as Equation (2).

(2) $$\frac{\partial w}{\partial t}=\frac{\partial w}{\partial H}\frac{\partial H}{\partial t}=-div\;J\;(2)$$

in which w is water content and t is time.

The relationship between the pore relative humidity and water content within concrete is described by sorption isotherm [9]. In the sorption isotherm, water content is a function of relative humidity. Thus, the terms of water content and flux are eliminated by substituting Equation (1) into Equation (2). If concrete is assumed to be a homogeneous material, a nonlinear moisture diffusion equation can be obtained for a one-dimensional problem as Equation (3).

(3) $$\frac{\partial H}{\partial t}=\frac{1}{\frac{\partial w}{\partial H}}div\left(C_{k}grad\;H\right)=div\left(D\;grad\;H\right)=D\frac{\partial^{2}H}{\partial x^{2}}\;$$

in which D is the moisture diffusion coefficient and defined as $kC_{k}$, and k is the reciprocal of slope of the sorption isotherm. When the slope of the sorption isotherm and permeability in the moisture diffusion equation is determined, the moisture diffusion coefficient is obtained. The moisture diffusion coefficient as a function of relative humidity is referred to as the moisture diffusion model.

The relative humidity on the exposed surface of concrete is generally higher than that in ambient air. To balance the relative humidity, the moisture is emitted from the surface. Thus, the boundary condition is expressed by correlation between the surface moisture and the humidity of ambient air as Equation (4).

(4) $$D{\left(\frac{\partial H}{\partial x}\right)}_{s}=s\left(H_{e}-H_{s}\right)$$

in which s is the surface factor, $H_{e}$ is the relative humidity of ambient air, and $H_{s}$ is the relative humidity on the exposed surface.

### 2.2. Finite Element Formulation

For finite element formulation, the relative humidity H(x,t) in solid medium is expressed as the product of the shape function and relative humidity at node as Equation (5).

(5) H(x,t)=[N(x)]{H(t)}

in which {H(t)} is the nodal humidity vector at a specific time, and [N(x)] is shape function vector.

The weak form of the moisture diffusion equation can be obtained using Galerkin method as Equation (6).

(6) $$\int_{\Omega }^{}{\left[N\right]}^{T}\left\{\frac{\partial H}{\partial t}-D\frac{\partial^{2}H}{\partial x^{2}}\right\}d\Omega =0$$

in which Ω is domain within element.

By applying boundary condition, the resulting finite element equation into global system is induced as Equation (7).

(7) $$\left[C\right]\left\{\frac{\partial H}{\partial t}\right\}+\left[K\right]\left\{H\right\}=\left\{Q\right\}$$

in which,

[C] = $\sum_{element}^{}\int_{\Omega }^{}{\left[N\right]}^{T}\left[N\right]d\Omega$; the moisture capacity matrix,

[K] = $\sum_{element}^{}\int_{\Omega }^{}{\left[B\right]}^{T}D\left[B\right]d\Omega +\sum_{element}^{}\int_{\Gamma }^{}f{\left[N\right]}^{T}\left[N\right]d\Gamma$; the diffusion matrix,

{Q} = $\sum_{element}^{}\int_{\Gamma }^{}sH_{e}{\left[N\right]}^{T}d\Gamma$; the moisture load vector,

$\left\{\frac{\partial H}{\partial t}\right\}$ is the derivative of relative humidity with respect to time,

B is the derivative of shape function,

Γ is boundary of element,

$H_{e}$ is ambient humidity,

s is surface factor.

Equation (7) is the ordinary differential equation which contains the derivative term with respect to time. The derivative term of relative humidity with respect to time can be expressed using the mean value theorem as Equation (8).

(8) $$\left\{\frac{\partial H}{\partial t}\right\}\approx \frac{\left\{H_{n+1}\right\}-\left\{H_{n}\right\}}{∆t}\;$$

in which ∆t is time step, $\left\{H_{n}\right\}$ is the nodal humidity vector at time $t_{n}$, and $\left\{H_{n+1}\right\}$ is the nodal humidity vector at time $t_{n+1}$.

Substituting Equation (8) into Equation (7) and rearranging Equation (7), an implicit form of algebraic equation for moisture diffusion analysis can be obtained as Equation (9).

(9) $$\left(\left[C\right]+∆t\left[K_{n+1}\right]\right)\left\{H_{n+1}\right\}=∆t\left\{Q_{n+1}\right\}+\left[C\right]\left\{H_{n}\right\}$$

in which $\left[K_{n+1}\right]$ is the diffusion matrix at time $t_{n+1}$ and $\left\{Q_{n+1}\right\}$ is the moisture load vector at time $t_{n+1}$.

Based on the finite element formulation described above, a moisture diffusion analysis code was developed using MATLAB [21]. The code was utilized for the generation of virtual experiment data of the moisture distribution in concrete and also for the inverse estimation of the moisture diffusion model.

## 3. Virtual Experiment of Moisture Diffusion of Concrete

**Figure 1:**
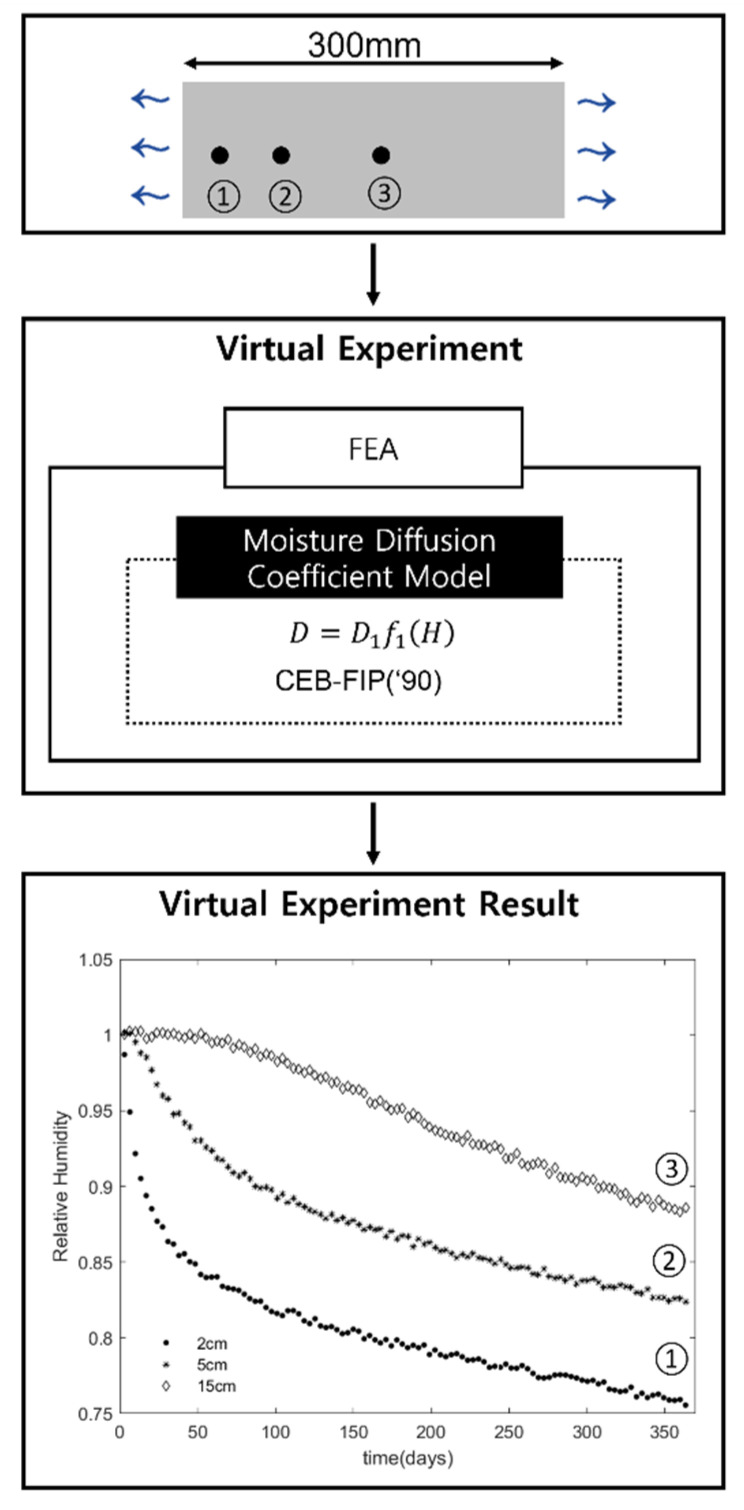
The schematic flow of the virtual experiment.

The virtual experiment in this study is to obtain the relative humidity distribution in concrete over time using a numerical simulation. The schematic flow of the virtual experiment is shown in Figure 1. The virtual experiment consists of creating an experimental model for simulation and conducting the finite element simulation based on the model.

**Figure 2:**
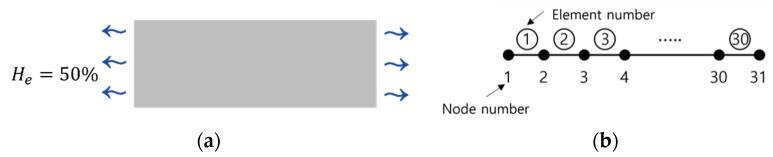
Virtual experiment model and finite element representation: (**a**) virtual model and (**b**) finite element representation.

The experimental model for the simulation is shown in Figure 2a. The model is 300 mm and one-dimensional configuration in which the moisture flows unidirectionally. Both sides of the boundary are exposed to ambient, and the moisture is being lost. Figure 2b shows finite element representation of the experimental model. The body of 300 mm in length was divided into 30 elements of 10 mm. The humidity data were collected at the nodes located 20 mm, 50 mm, and 150 mm away from the boundary. The humidity data collected at the nodes through the virtual experiment are referred to as observation data in this study.

For the virtual experiment shown in Figure 1, the moisture diffusion model D provided by CEB-FIP(’90) [22] was used. In CEB-FIP(’90), the moisture diffusion model is expressed as a function of the pore relative humidity under isotherm condition as shown in Equation (10) [23].

(10) $$D=D_{1}f_{1}\left(H\right)$$

in which $D_{1}$ is the moisture diffusion coefficient in saturated condition and calculated by Equation (11), and $f_{1}\left(H\right)$ is a function reflecting pore relative humidity and calculated by Equation (12).

(11) $$D_{1}=\frac{D_{1,0}}{f_{ck}/f_{ck0}}$$

in which $D_{1,0}$ = $3.6\times 10^{-6}$($m^{2}/h$), $f_{ck0},\;f_{ck}$ are parameters related to compressive strength.

(12) $$f_{1}\left(H\right)=\left[\alpha +\frac{1-\alpha }{1+{\left[\left(1-H\right)/\left(1-H_{c}\right)\right]}^{n}}\right]\;$$

in which $\alpha =D_{0}/D_{1}$, $D_{0}$ is the moisture diffusion coefficient in completely drying condition, $H_{c}$ is the pore relative humidity at $0.5D_{1}$, and n is the parameter depending on concrete mixture of concrete.

Two virtual materials, M1 and M2, were used to generate the virtual experiment data. The model parameters for the material M1 and M2 are shown in in Table 1. Both M1 and M2 have the same parameters except for the parameter n.

For the boundary conditions shown in Equation (4), the ambient humidity $H_{e}=50\%$ and the surface factor s is calculated using Equation (13), which is suggested by Sakata [12].

(13) $$s=2.17\times 10^{-3}\left(W/C\right)-8.56\times 10^{-4}$$

in which W/C is water to cement ratio. W/C=50% was used to calculate the surface factor s.

The relative humidity distribution with time was calculated by the finite element simulation using the virtual materials, and the final virtual experiment data were generated by adding a random noise to the relative humidity distribution data. The random noise was introduced by using Equation (14).

(14) $$\bar{H}=H^{sim}+\omega \delta_{0}$$

in which $\bar{H}$ is the final virtual experiment data, $H^{sim}$ is the simulated humidity without noise, ω is a uniformly distributed random variable from −1 to 1, and $\delta_{0}$ is 0.3% of the maximum value of the simulated humidity data.

**Figure 3:**
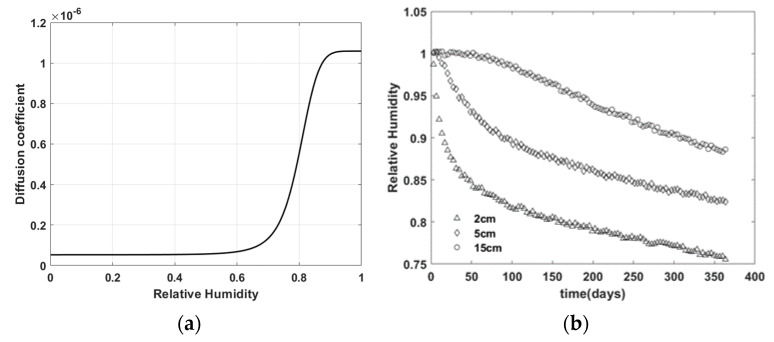
Virtual experiment data with material M1—(**a**) moisture diffusion model D; (**b**) relative humidity distribution over time.

**Figure 4:**
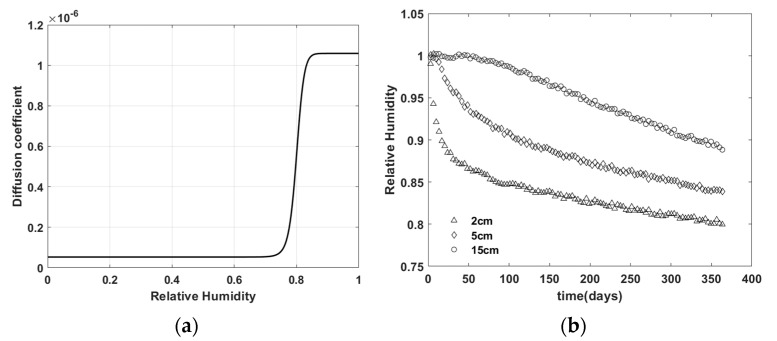
Virtual experiment data with material M2—(**a**) moisture diffusion model D; (**b**) relative humidity distribution over time.

Figure 3 and Figure 4 shows the virtual experiment data generated by the finite element simulation using material M1 and M2, respectively.

## 4. Inverse Estimation of Moisture Diffusion Model

### 4.1. Artificial Neural Network

An artificial neural network (ANN) can learn from data to map nonlinear relationships between input and output data. ANN is mainly used as a tool for classification or regression by learning rules from training data sets [24]. In this study, ANN was used as a moisture diffusion model for concrete. The parameters defining the ANN are inversely estimated using global response of the moisture diffusion of concrete, which is a moisture distribution in concrete over time.

**Figure 5:**
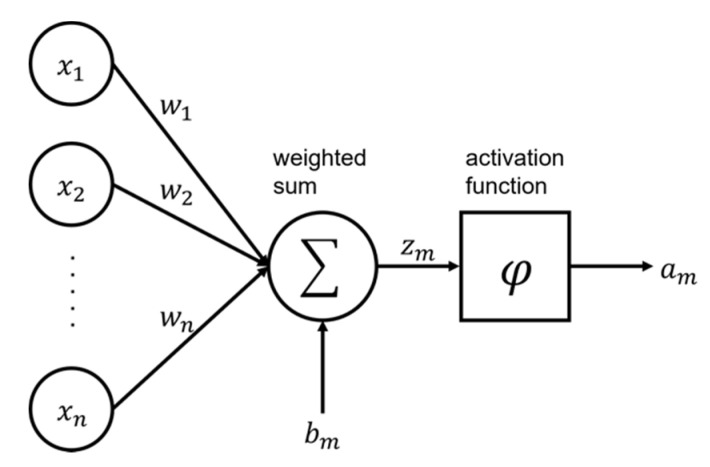
An output computation at a single node.

ANNs consist of an input layer, hidden layers, and an output layer, and each layer contains computation nodes. The nodes in each layer are fully connected with the nodes in the previous and the next layer. Each node in the hidden layers is given input values from the nodes in the previous layer. The input values are summed up with weight and bias using Equation (15) and are activated through a nonlinear activation function using Equation (16). The computation for the activated output at a single node is illustrated in Figure 5.

(15) $$z_{m}=\overset{n}{\underset{i=1}{\sum }}x_{i}w_{i}+b_{m}$$

in which $z_{m}$ is the weighted sum values from input value in the node, $x_{i}$ is the input value in mth layer, $w_{i}$ is weight between (m−1)th layer and mth layer, $b_{m}$ is bias between (m−1)th layer and mth layer, and n is the number of nodes in (m−1)th layer.

(16) $$a_{m}=\varphi \left(z_{m}\right)\;$$

in which $a_{m}$ is the output value of given node in m-th layer and φ is activation function.

**Figure 6:**
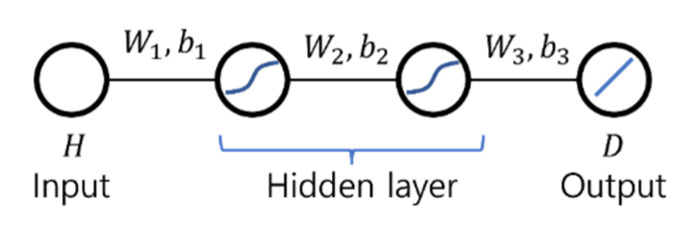
Artificial Neural Network N1.

**Figure 7:**
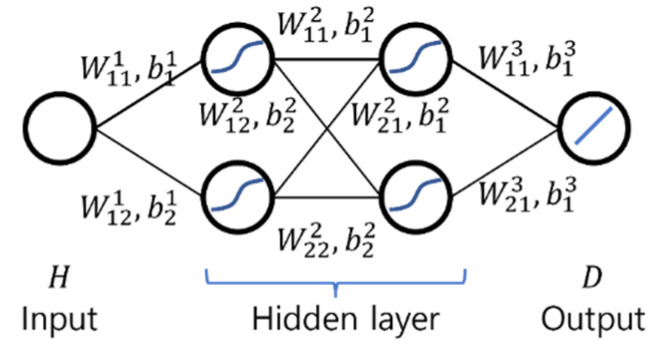
Artificial Neural Network N2.

Two ANNs of simple architecture were used as moisture diffusion model as shown in Figure 6 and Figure 7. Both networks N1 and N2 have an input layer, two hidden layers, and an output layer. The networks N1 and N2 have one node for humidity H in input layer and one node for moisture diffusion coefficient D in output layer. The network N1 has one node in each hidden layer while N2 has two nodes. For the activation function of N1 and N2, hyperbolic tangent function (tanh) was used for the nodes in hidden layers and linear function for the nodes in output layer.

Usually, an ANN is trained through the iterative forward and backward propagation. Forward propagation is a process in which ANN outputs the predicted value through operations in the hidden layer while backward propagation is a process that adjusts the weight and bias for minimizing difference between the known target value and predicted value. There are many algorithms for backward propagation such as gradient descent, resilient propagation, and so on [25].

In order to train an ANN by the forward-backward propagation, existing known pairs of input and output data are required. However, the pairs of the input and output data do not exist in this study, hence the forward–backward propagation could not be used to train the ANN as moisture diffusion model. Instead, the global moisture diffusion response of concrete, which is the humidity distribution over time, is provided by the virtual experiment. Using the humidity distribution data and a finite element analysis, the ANN for moisture diffusion model was inversely estimated. The inverse estimation is an optimization process of the ANN model so as to minimize the difference between the global moisture diffusion response calculated by a finite element analysis using the ANN as a moisture diffusion model and the data provided by the virtual experiment.

### 4.2. Optimization for the Inverse Estimation of Moisture Diffusion Model

**Figure 8:**
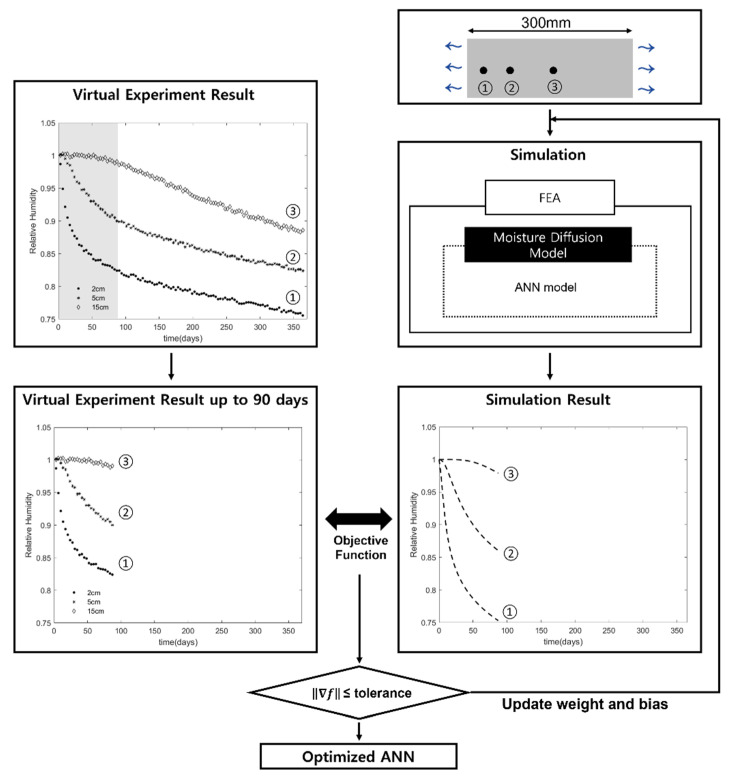
The schematic flow of inverse estimation.

Figure 8 shows a schematic flow of the inverse estimation of moisture diffusion model using the virtual experimental data. The procedure for the inverse estimation is as follows.

STEP1: to initialize the ANN model. The weights and biases of the ANN were initialized by random values between 0 and 1. At this point, the initialized ANN model maps an arbitrary relationship between humidity and moisture diffusion coefficient.

STEP2: finite element simulation using the ANN as a moisture diffusion model. The geometry and boundary condition of the finite element model are identical to those used in the virtual experiment. The relative humidity data at the observation locations identical to those in experiment are extracted from the finite element simulation result.

STEP3: to adjust the weights and biases in direction to minimize the error between the humidity data from the finite element simulation using the ANN model and the humidity data observed from the experiment. To minimize the error, as an optimization method, Interior Point Method (IPM), was used with the objective function shown in Equation (17).

(17) $$f\left(W,b\right)=\sqrt{\frac{1}{N}\frac{1}{P}\overset{N}{\underset{i=1}{\sum }}\overset{P}{\underset{j=1}{\sum }}{\left({\bar{H}}_{ij}^{}-H_{ij}\left(W,b\right)\right)}^{2}}$$

in which f is the objective function, W and b are respectively weights and biases of the ANN model, N is the number of investigated points, P is the number of time step for observation, and $H_{ij}\left(W,b\right)$ is the relative humidity calculated by finite element simulation with the updated ANN model.

ITERATION: to repeat STEP2 and STEP3 until the gradient of the objective function falls below a tolerance. The tolerance of $1\times 10^{-6}$ was used for this study.

IPM is a type of optimization algorithm that finds the minimum of a nonlinear multivariable function with constraints such as equalities, inequalities, and the range of variables. The feasible region is set by the given constraints and, in that region, the optimization process is performed by iterative calculation [26,27]. For optimizing the objective function, the IPM algorithm in *fmincon* function provided by MATLAB was employed [21]. In the process of optimization, the constraint of equality and inequality was not set, but only the constraint of the variable range. The initial condition of variables for optimization was set an arbitrary random value between 0 and 1, and the constraint of the range of variables was set to −300 as a lower limit and 300 as an upper limit.

To test the performance of the methodology for the inverse estimation, two materials (M1, M2), two ANN architectures (N1, N2), and two observation periods (90 days, 180 days) were considered. The labels of the test cases are summarized in Table 2.

**Figure 9:**
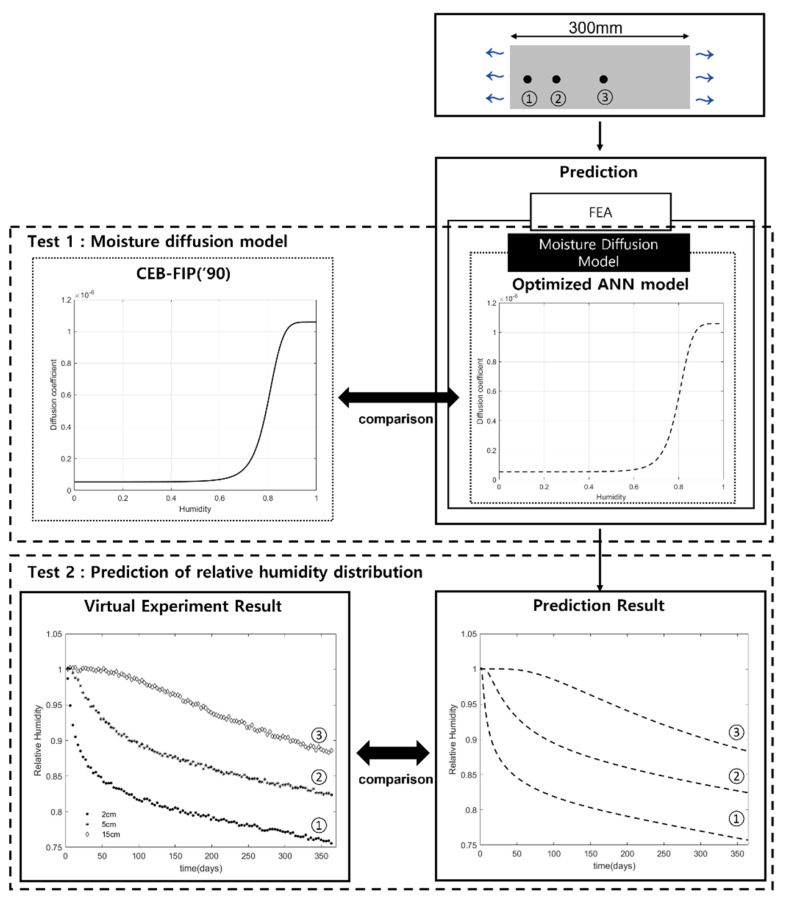
The schematic flow of the performance test.

The performance test was conducted in two ways, as shown Figure 9. Test 1 was to compare the inversely estimated ANN model with the moisture diffusion model used in the virtual experiment. Test 2 was to compare the long-term relative humidity data simulated using the ANN with those from the virtual experiment.

## 5. Result and Discussion

### 5.1. Result

**Figure 10:**
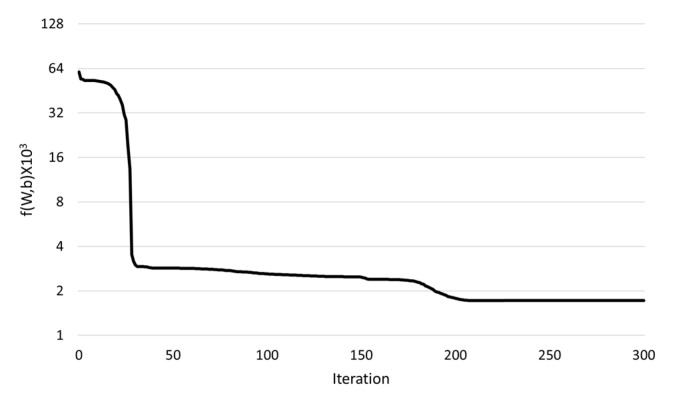
An example of convergence of objective function with iteration during optimization—Network N1, Material M2, and observation period of 180 days.

Figure 10 shows an example of the convergence of objective function over iterations. The objective function calculated through Equation (17) is the error between humidity distribution from the virtual experiment data during the observation period and that calculated from the finite element simulation using the ANN. The value of the objective function decreased as the iterations increased during the optimization process. In particular, it decreased rapidly around the iteration numbers of 1, 30, and 180. After iteration number of 210, no further decrease in objective function was found.

**Figure 11:**
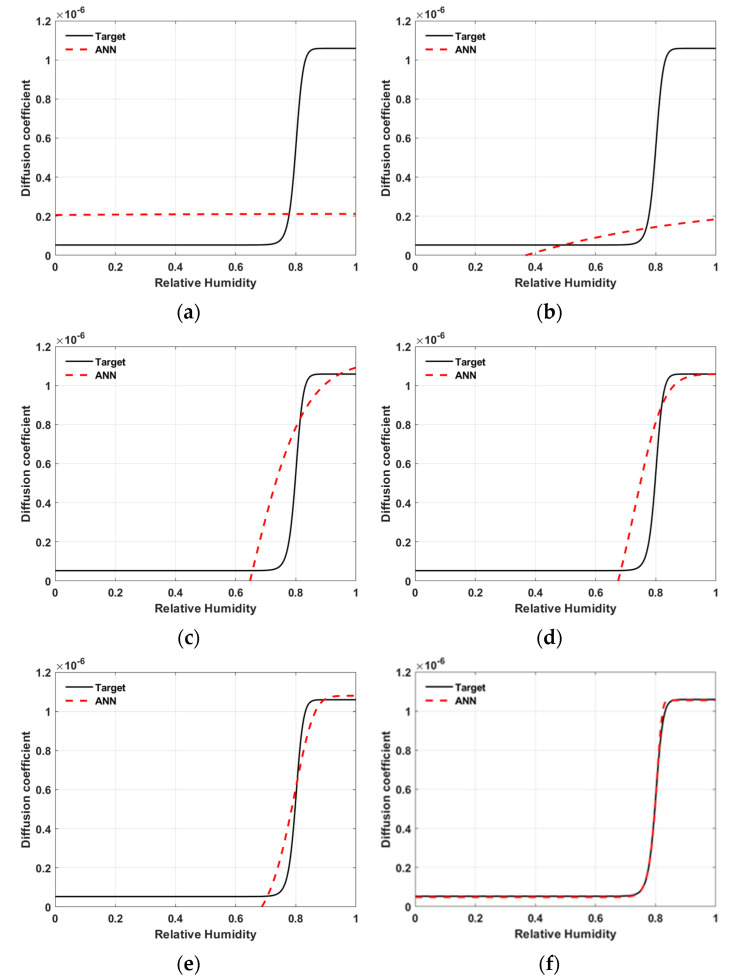
An example of adaptive process with iteration in optimization (Network N1, Material M2, and observation period of 180 days)—the number of the iterations: (**a**) 0, (**b**) 20, (**c**) 100, (**d**) 150, (**e**) 200, (**f**) 300.

Figure 11 shows the adaptation process of the ANN model over iterations during optimization. The solid line is the target model, i.e., the moisture diffusion model D in Equation (10) used for the virtual experiment. The dashed line is the inversely estimated ANN model. The ANN model is being fitted to the target model with increasing iterations during the optimization process.

**Figure 12:**
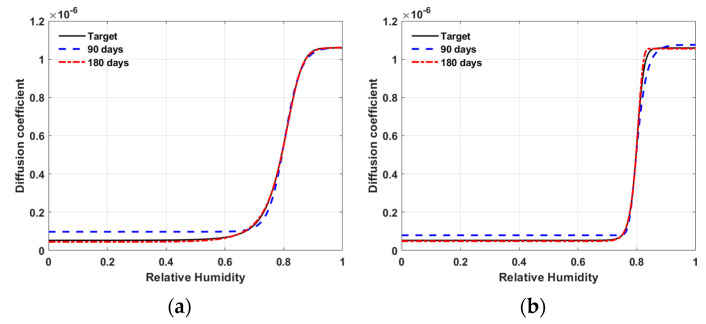
Results of inverse estimation using N1—target model versus ANN model (**a**) material M1; (**b**) material M2.

Figure 12 shows the inversely estimated ANN model of N1 after 300 of iterations. The solid line is the target model. The dashed line and dotted line are the inversely estimated ANN model using the observation data up to the 90 days and 180 days, respectively. As shown in the figure, the inversely estimated ANN models with the observation period of 180 days rather than 90 days show better fits to the target models for both material M1 and M2.

**Figure 13:**
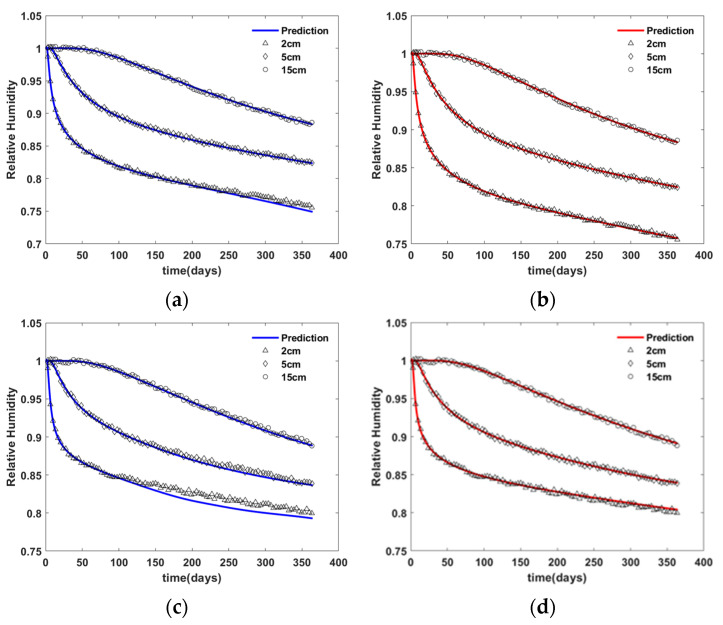
Comparison of virtual experimental data with the predicted data based on ANN N1—(**a**) material M1 and 90 day observation, (**b**) material M1 and 180 day observation, (**c**) material M2 and 90 day observation, and (**d**) material M2 and 180 day observation.

Figure 13 shows the relative humidity predicted by finite element simulation using the inversely estimated ANN model of N1 (solid lines) and virtual experiment data (markers). As seen in the figure, the prediction with the observation period of 180 days rather than 90 days fits the experiment better. Note that the prediction was conducted up to 360 days. The root mean square (RMS) of the difference between the prediction using the ANN and virtual experiment data is shown in Table 2.

**Figure 14:**
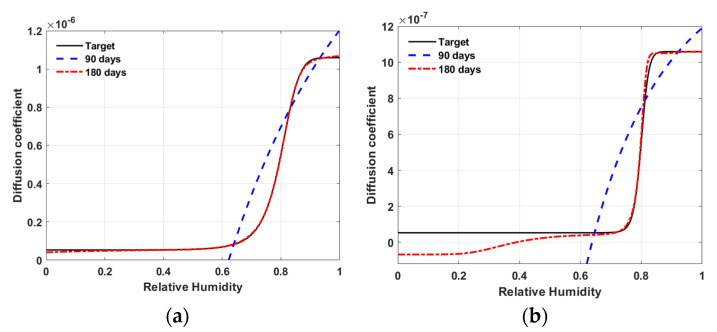
Results of inverse estimation using N2—target model versus ANN model (**a**) material M1; (**b**) material M2.

Figure 14 shows the inversely estimated ANN model of N2 after 300 iterations. The ANN models with the observation period of 90 days (dashed lines) could not fit well with the non-linear variation of the target model for either material M1 or M2. The ANN models with the observation period of 180 days (dotted lines) fit the target model well over the entire humidity range for material M1, but only fit the target model over the humidity range above 0.6 for material M2.

**Figure 15:**
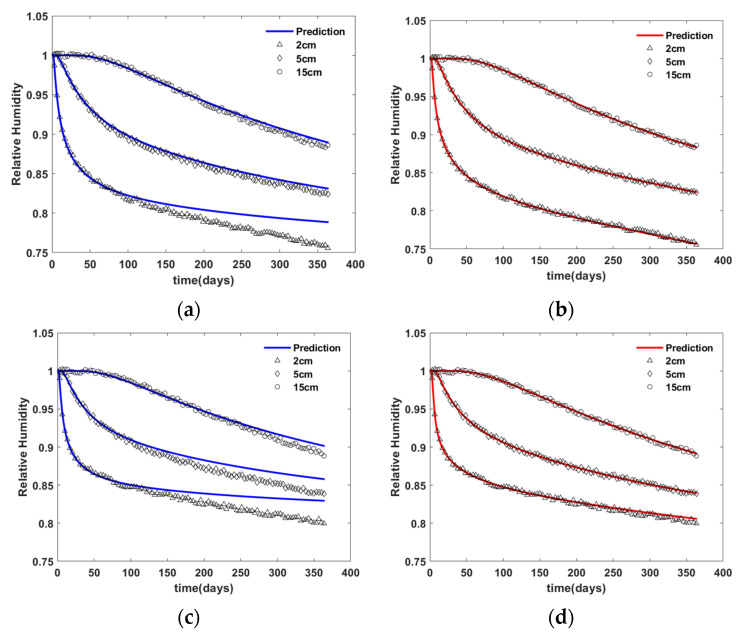
Comparison of virtual experimental data with the predicted data based on ANN N2—(**a**) material M1 and 90 day observation, (**b**) material M1 and 180 day observation, (**c**) material M2 and 90 day observation, and (**d**) material M2 and 180 day observation.

Figure 15 shows the relative humidity predicted by finite element simulation using the inversely estimated ANN model of N2 (solid lines) and virtual experiment data (markers). As seen in the figure, the prediction using the ANN model with observation period of 180 days fits the experiment well over the entire prediction range. However, the predictions using the ANN model with observation period of 90 days only fit the experiment well up to 90 days. The root mean square (RMS) of the difference between the prediction using the ANN models and virtual experiment data is shown in Table 3.

### 5.2. Discussion

This study showed that a highly nonlinear moisture diffusion model can be inversely estimated using global response of moisture diffusion behavior of concrete. For the inverse estimation, ANNs of simple architecture were used as a moisture diffusion model. No direct information regarding the shape of the moisture diffusion model used for the generation of the virtual experiment data was used for the inverse estimation.

The ANN model of “simple” architecture is actually not simple. Equation (18) shows an explicit representation of the feedforward process of the ANN model of N1 shown in Figure 5 that computes the output D using the input H.

(18) $$D=w_{3}\;\tan h(w_{2}\;\tan h(w_{1}H+b_{1})+b_{2})+b_{3}\;$$

in which $w_{i}$ and $b_{i}$ are the weights and biases of the ANN model of N1, and I = 1, 2, and 3.

Equation (18) has six parameters defining the ANN model and the hyperbolic tangent (tanh) was used as an activation function, i.e., the equation has enough complexity to learn the shape of the moisture diffusion model used in the virtual experiment. Therefore, even an ANN of “simple” architecture can be used for the inverse estimation of the moisture diffusion t model for concrete material.

If an ANN model has sufficient complexity to learn the moisture diffusion model used in the virtual experiment, a relatively simpler ANN model is advantageous for the inverse estimation. The ANN model of N1 has six parameters (three weights and three biases). On the other hand, the ANN model of N2 has 16 parameters (eight weights and eight biases). The inverse estimation result of the ANN model of N1 shown in Figure 6 fits the target better than that of the ANN model of N2 shown in Figure 7. This seems to be because the ANN model with a more complex structure is more likely to fall into a local minimum during the minimizing the objective function in the inverse estimation process.

The quality of the inverse estimation is dependent on the humidity data range used for the inverse estimation. This statement can be validated with the result of the inversely estimated ANN model of N2 for material M1 shown in Figure 14a. The ANN model with the observation period of 90 days (dashed lines) could not fit well the non-linear variation of the target model, but the ANN model with the observation period of 180 days (dotted lines) fit the target model well. The difference of observation period of 90 days and 180 days means not just the number of the data points but the relative humidity range of the data used for the inverse estimation.

**Figure 16:**
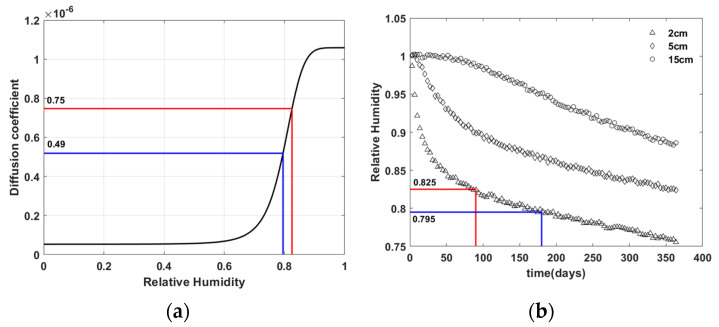
Relative humidity range corresponding to observation period in virtual experiment with material M1—(**a**) moisture diffusion model D; (**b**) relative humidity distribution over time.

Figure 16 shows the relative humidity ranges corresponding to the observation periods used for the virtual experiment with material M1. The lowest relative humidity observed in the virtual experiment up to 90 days is 0.825 (Figure 16b) that corresponds to the diffusion coefficient of $0.75\times 10^{-6}$ (Figure 16a). It means that the virtual experiment data of 90 day observation period provides implicit information regarding the relative humidity vs. moisture diffusion coefficient relationship in the range of relative humidity above 0.825 and the diffusion coefficient above $0.75\times 10^{-6}$. This range covers only about 30% of the upper part of the total relative humidity vs. moisture diffusion coefficient relationship. It means that the ANN model had a chance to access only about 30% of the relative humidity vs. moisture diffusion coefficient relationship during the inverse estimation, making it difficult to inversely estimate the model.

On the other hand, the lowest relative humidity observed in the virtual experiment up to 180 days is 0.795 (Figure 16b), which corresponds to the diffusion coefficient of $0.49\times 10^{-6}$ (Figure 16a). It means that the virtual experiment data of 180-day observation period provide the implicit information regarding the relative humidity vs. diffusion coefficient relationship in the range of relative humidity above 0.795 and the diffusion coefficient above $0.49\times 10^{-6}$. This range covers about 60% of the upper part of the total relative humidity–moisture diffusion coefficient relationship. Therefore, the ANN model had a chance to access about 60% of the relative humidity vs. moisture diffusion coefficient relationship during the inverse estimation, making it relatively easy to inversely estimate the model.

A longer observation period is advantageous for the inverse estimation of the ANN model. However, even with the observation period of 180 days the lowest relative humidity provided by the virtual experiment is 0.795, i.e., the virtual experiment does not provide humidity information in range lower than 0.795. Despite this fact, the inversely estimated ANN model shown in Figure 14a is well matched with the target in entire range of relative humidity. Although the virtual experiment data themselves do not provide relative humidity information in the range lower than 0.795, since the ambient relative humidity was set to 0.5 as a boundary condition, there will be some locations near the surface of the concrete specimen with a relative humidity of less than 0.795.

In the inverse estimation process of the ANN model, a finite element analysis is conducted and the global diffusion matrix [K] shown in Equation (7) is calculated. To calculate a portion of the global diffusion matrix [K] corresponding to a node, the feedforward process of ANN model should be performed with the relative humidity of the node as an input. At this point, the feedforward process of ANN model is performed with a relative humidity of less than 0.795, and the ANN model returns a moisture diffusion coefficient as output. During the early stages of the inverse estimation process, the moisture diffusion coefficient corresponding to a humidity of less than 0.795 may not match the target. However, over iterations, the weights and biases of the ANN model are updated in the direction to minimize the difference between the relative humidity distribution calculated from finite element analysis and those provided by virtual experiment. This means that the ANN model should be close to the target including the lower part of the target in order to minimize the difference.

## 6. Conclusions

This study demonstrated that a moisture diffusion behavior of concrete can be inversely estimated with an ANN of simple architecture using global moisture distribution data. Based on the research results, the conclusions are as follow:

- A simple architecture of ANN has sufficient complexity to learn the moisture diffusion behavior of concrete defined by CEB-FIP(’90) model.
- An ANN with a simpler architecture gives a better result in the inverse estimation of moisture diffusion behavior since it is less likely to stay at local minimum in the optimization process.
- Moisture distribution data should cover enough range of relative humidity for the inverse estimation of an ANN moisture diffusion model.
- A longer observation period gives a better result in the inverse estimation of an ANN moisture diffusion model since it provides a wider range of relative humidity information.
- A long-term moisture distribution in concrete can be predicted using an ANN moisture diffusion model inversely estimated using short-term global response.

The methodology shown in this study should be validated using laboratory or field test data in the future. It can be applied to a structural analysis that requires long-term relative humidity distribution prediction using short-term humidity data measured in the concrete. The methodology can help to understand new concrete materials with unknown moisture diffusion behavior.

## Figures and Tables

**Table 1:** The parameters of the moisture diffusion model for virtual experiments.

Material	$D_{1}$			f(H)		
	$D_{1.0}\;(m^{2}/h)$	$f_{ck0}\;(\mathrm{MPa})$	$f_{ck}\;(\mathrm{MPa})$	α	$H_{c}$	n
M1	3.6 × 10^−6^	10	34	0.05	0.80	6
M2						16

**Table 2:** Labels of performance test for inverse estimation.

Label	Neural Network Architecture	Material	Observation Period
N1M1D90	N1	M1	90 days
N1M1D180	N1	M1	180 days
N1M2D90	N1	M2	90 days
N1M2D180	N1	M2	180 days
N2M1D90	N2	M1	90 days
N2M1D180	N2	M1	180 days
N2M2D90	N2	M2	90 days
N2M2D180	N2	M2	180 days

**Table 3:** RMS of the predicted data based on ANN model with respect to the virtual experimental data.

	N1		N2	
	M1	M2	M1	M2
90 days	0.0049	0.0119	0.0221	0.0289
180 days	0.0029	0.0035	0.003	0.0039

## Data Availability

Not applicable.
